# Knowledge mapping of freezing of gait in Parkinson’s disease: a bibliometric analysis

**DOI:** 10.3389/fnins.2024.1388326

**Published:** 2024-09-09

**Authors:** Yue Jiao, Zaichao Liu, Juan Li, Yan Su, Xianwen Chen

**Affiliations:** ^1^Department of Neurology, The First Affiliated Hospital of Anhui Medical University, Hefei, China; ^2^Department of Neurology, The Second Affiliated Hospital of Anhui Medical University, Hefei, China

**Keywords:** Parkinson’s disease, freezing of gait, bibliometric analysis, hotspots, VOSviewer, CiteSpace

## Abstract

**Objective:**

Among the disturbing motor symptoms in Parkinson’s disease (PD), freezing of gait (FOG) stands out as one of the most severe challenges. It typically arises during the initiation of gait or when turning. This phenomenon not only impose a heavy burden on patients, but also on their families. We conduct a bibliometric analysis to summarize current research hotspots and trends concerning freezing of gait in Parkinson’s disease (PD-FOG) over past two decades.

**Methods:**

We retrieved articles and reviews published in English about PD-FOG in the Web of science Core Collection database from 2000 to 2023 on November 30,2023. The tools VOSviewer and CiteSpace facilitated a visual analysis covering various aspects such as publications, countries/regions, organizations, authors, journals, cited references, and keywords.

**Result:**

This study includes 1,340 articles from 64 countries/regions. There is a growth in publications related to PD-FOG over the past two decades, maintaining a stable high output since 2018, indicating a promising research landscape in the field of PD-FOG. The United States holds a leading position in this field, with Nieuwboer A and Giladi N being two of the most influential researchers. Over the past two decades, the research hotspots for PD-FOG have primarily encompassed the kinematic characteristics, diagnosis and detection, cognitive deficits and neural connectivity, as well as therapy and rehabilitation of PD-FOG. Topics including functional connectivity, virtual reality, deep learning and machine learning will be focal points of future research.

**Conclusion:**

This is the first bibliometric analysis of PD-FOG. We construct this study to summarize the research in this field over past two decades, visually show the current hotspots and trends, and offer scholars in this field concepts and strategies for subsequent studies.

## 1 Introduction

Freezing of gait (FOG) is defined as “a brief, episodic absence or marked reduction of forward progression of the feet despite the intention to walk” (Giladi and Nieuwboer, 2008). Among gait disorders in Parkinson’s disease (PD), it is considered one of the most impairing, affecting 80% of people with severe PD (Kwok et al., 2022). FOG leads to falls in patients with PD, which not only in turn contributes to immobility, loss of independence, increased injury concern, quality of life impairment (De Boer et al., 1996; Moore et al., 2007; Walton et al., 2015b), but also increases burden on caregivers (Pressley et al., 2003; Schrag et al., 2006). Thus, freezing of gait in Parkinson’s disease (PD-FOG) has drawn substantial attention from medical scholars and professionals.

Due to the dangers of FOG, its early prediction and screening is necessary. In addition to screening with conventional scales, like Freezing of Gait Questionnaire (FOG-Q), wearable devices provide some new ideas in this field (Tripoliti et al., 2013; Mancini et al., 2021; Zhang et al., 2024). Recently, electroencephalography has been found that may be able to predict FOG before it occurs (Handojoseno et al., 2015). Management for PD-FOG include drug treatment, exercise trainings, deep brain stimulation and noninvasive brain stimulations such as repetitive transcranial magnetic stimulation (rTMS) and transcranial direct current stimulation (tDCS) (Nonnekes et al., 2015; Gao et al., 2020; Rahimpour et al., 2021). Although research on PD-FOG has seen a gradual increase in recent years, there remains a lack of research to investigate the current hotspots and future trends in this field, which is crucial for providing indispensable knowledge for clinical endeavors and supporting the evolution of subsequent studies.

Bibliometrics is an interdisciplinary methodology by applying mathematical and statistical methods for the quantitative analysis of literature in specific research field (Thompson and Walker, 2015). Utilizing literature analysis tools such as CiteSpace and VOSviewer helps scholars uncover advanced developments and trends in specific research areas (van Eck and Waltman, 2010; Chen, 2017).

In this study, CiteSpace and VOSviewer were utilized to carry out a bibliometric and visual analysis of literature on PD-FOG from the recent 20 years, involving the extraction and examination of data concerning publications, countries/regions, organizations, journals, authors and keywords. We anticipate that our findings will facilitate a better comprehension of the research status within this domain for upcoming researchers and foster the development of new research ideas.

## 2 Materials and methods

### 2.1 Data source and search strategy

**Figure 1:**
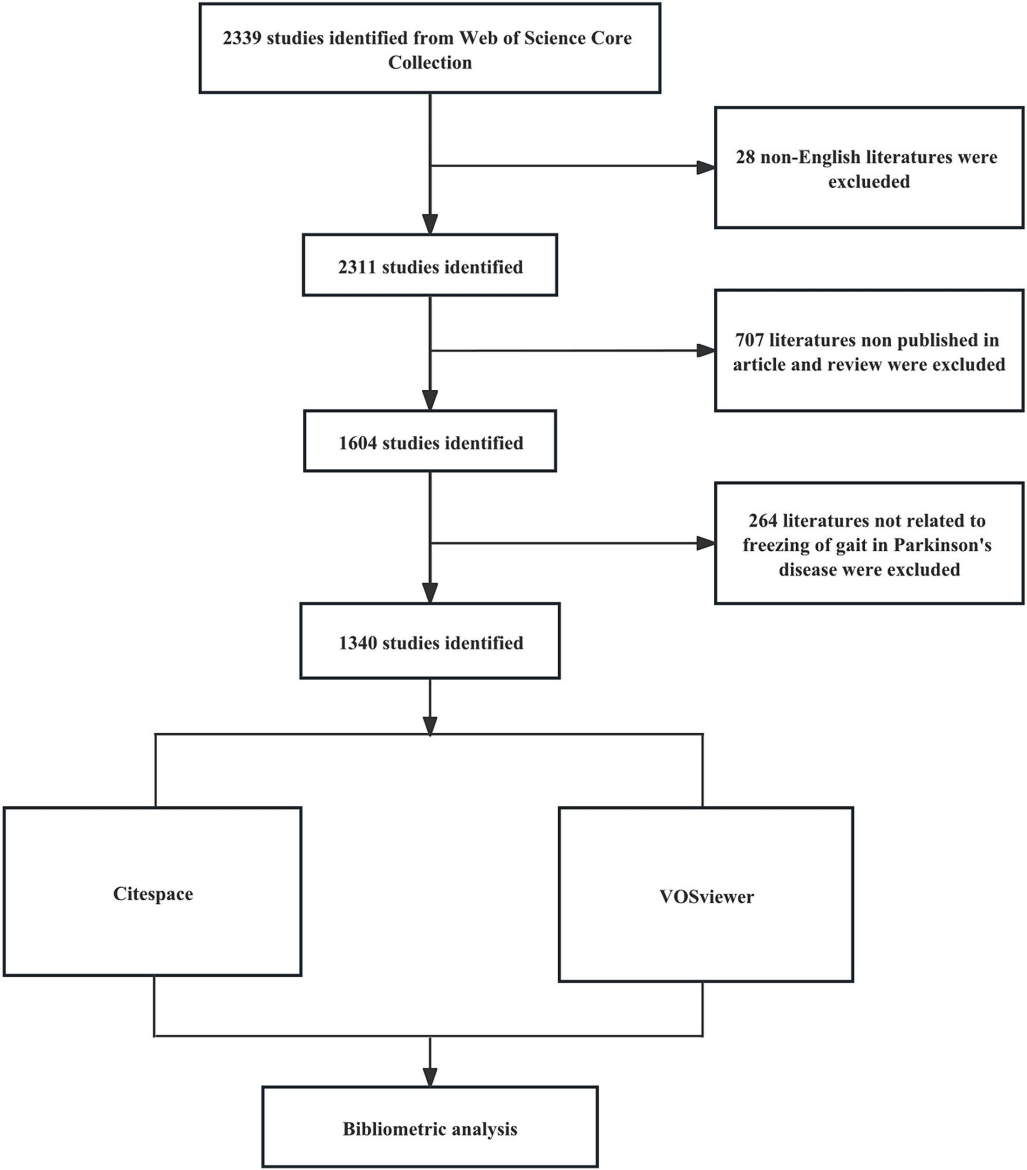
Flow chart of literature retrieval and analysis methods.

A comprehensive literature search was conducted on the Web of Science Core Collection (WOSCC) database, on November 30, 2023. The search formula was [TS = (“Parkinson Disease” OR Parkinson* OR “PD”) AND TS = (“freezing of gait” OR “gait freezing” OR “freezing gait” OR “frozen gait” OR “gait arrest” OR “FOG”)] AND [Language = (English)]. Spanning from 2000 to 2023, the research strictly included articles and review articles, omitting all other document formats. We reviewed the title and abstract of each publication in detail, excluding articles that were irrelevant to the topic. Finally, we collected 1,340 articles that matched the criteria, provided in the formats of ‘full record and cited references’ and “plain text’. Figure 1 shows the detailed process.

### 2.2 Analysis

VOSviewer V1.6.20 and CiteSpace V6.2.R4 Advanced are two common software tools for constructing and visualizing bibliometric networks maps. In these maps, nodes denote items like countries/regions, organizations and journals. The size and color of these nodes correspond to the quantity and classification of these items, respectively. The lines between nodes indicate the level of collaboration or co-citation among these items. Utilizing VOSviewer, we gathered information about countries/regions, organizations, co-authorship, journals, citation, co-citation, and keywords to construct network maps, respectively. Utilizing CiteSpace for analysis of publication, co-cited reference, citation bursts, keyword bursts, as well as the dual-map overlay of journals, involved settings such as a 2000–2023 timespan, yearly increments per slice, scale factor (k) of 25, and a selection of the top 50 entities (Top *N* = 50) for inclusion. The extraction of cluster labels was performed using the log-likelihood ratio (LLR) algorithm, with all other software parameters remaining at their default values. The dual-map journals overlay reveals the range of disciplines among journals focusing on PD-FOG research. The map displays clusters of citing journals on the left and cited journals on the right, with labels that denote their research fields. Colored lines connecting these clusters represent the citation trajectories from the citing journals to the cited ones.

Besides, we use Excel 2021 to analyze the annual publications. The data for journal impact factors (IF) and their categorization within the Journal Citation Reports (JCR) were collected from the Web of Science, dated December 9, 2023, for this study.

## 3 Results

### 3.1 Quantitative analysis of publication

**Figure 2:**
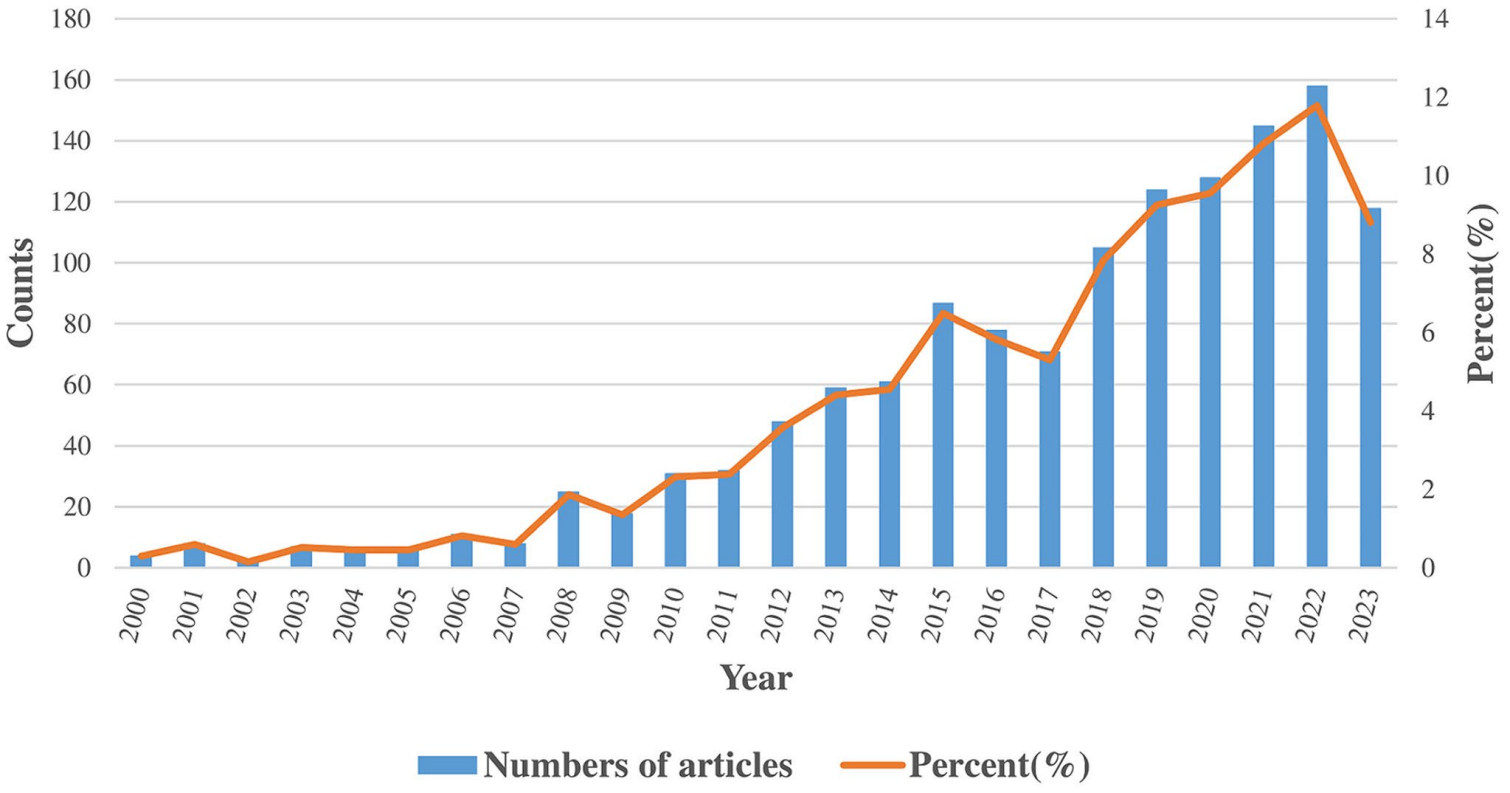
The annual number and trend of publications about PD-FOG.

Our study gathered 1,340 publications, consisting of 1,163 articles and 177 reviews. The trends in annual distribution are depicted in Figure 2. Between 2000 and 2023, the annual number of publications has shown a general upward trend. According to the quantity of publications, the period is divided into three phases. In the initial phase (2000–2007), there were notably low yearly publication counts (less than 10 per year), highlighting the nascent stage of research in this field. In the second phase (2008–2017), the field experienced its first significant increase in publication volume, with an annual average of more than 50 publications. The total publications in this phase were nearly 10 times that of the first phase. In the third phase (2018–2023), research in the field experienced an academic boom. The annual publication output is over 100, and the total publications exceeds the cumulative output of the preceding phases.

### 3.2 Analysis of countries/regions and organizations

**Figure 3:**
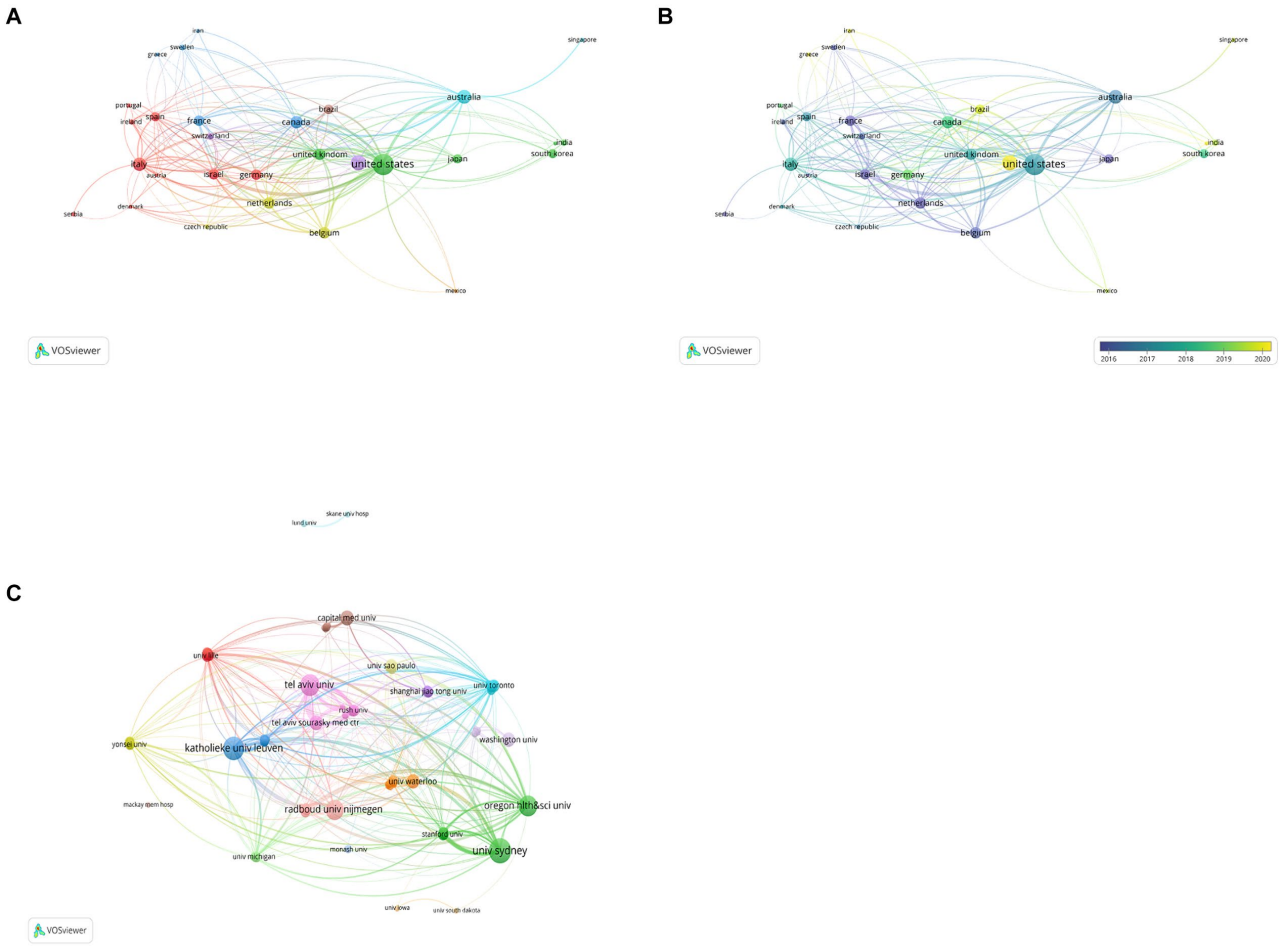
Analysis of countries/regions and organizations involved in studies related to PD-FOG, with a minimum number of publications of five. **(A)** The co-occurrence and clustering map of countries/regions. **(B)** The overlay visualization map of countries/regions. **(C)** The co-occurrence and clustering map of organizations involved in studies related to PD-FOG.

Articles from 64 countries/regions and 1,748 organizations contributed to the research progress of PD-FOG. Table 1 shows, among the top 10 countries/regions based on the number of publications, the United States ranked first (*n* = 37,527.99%), followed by China (*n* = 18,413.73%) and Australia (*n* = 14,010.45%). Publications from these three countries comprised 53.6% of the aggregate number of articles retrieved. Except China, the top 10 countries are all developed countries, indicating developed countries have made great efforts in the development of the field of PD-FOG. Figure 3A further depicts the network of collaboration between countries/regions with a publication count exceeding five, with active cooperation relationships especially evident between the United States, Australia, Israel, and Canada. In terms of citations, Israel, although having fewer publications, recorded the greatest mean citations per article compared to other countries/regions, establishing positive collaborative relationships with other countries and propelling the early development of this field (Figure 3B). In contrast, China, with its substantial volume of publications (ranking second among all countries), showed relatively lower total and average citation counts. It suggests that China should pay more attention to strengthen the monitoring of research quality, promote better alignment of research topics with international hotspots, and actively seek international partners while ensuring the number of studies.

**Table 1:** The top 10 countries/regions with the most publications.

Rank	Country/region	Documents	Count (%)	Citations	Average citation
1	The United States	375	27.99	16,706	44.55
2	China	184	13.73	2,437	13.24
3	Australia	140	10.45	7,327	52.34
4	Italy	134	10	4,965	37.05
5	Canada	117	8.73	2,812	24.03
6	The United Kingdom	100	7.46	5,646	56.46
7	Netherlands	96	7.16	6,258	65.19
8	Belgium	92	6.87	6,424	69.83
9	Israel	83	6.19	8,949	107.82
10	Germany	82	6.12	2,388	29.12

The top 10 organizations with largest publication volume are University of Sydney, Katholieke Universiteit Leuven, Tel Aviv University, Oregon Health and Science University, Radboud Universiteit Nijmegen, Capital Medical University, Tel Aviv Sourasky Medical Center, University of Waterloo, University of Washington, Universidade de São Paulo (Table 2). A total of six of these organizations are located in the Netherlands, Israel and the United States. Tel Aviv University and Katholieke Universiteit Leuven both exhibit strong performance in terms of publication output and average citations received. This means that they are leading organizations in this field, with their research being of high quality and impact, thereby advancing the field of PD-FOG. By analyzing the cooperation networks between organizations with a minimum number of publications of five, we further reveal how these leading organizations strengthen their influence in the field through extensive cooperation networks (Figure 3C). Figure 3C show that Katholieke Universiteit Leuven largely cooperated with Oregon Health and Science University, Tel Aviv University and University of Sydney, and Radboud Universiteit Nijmegen. University of Lille, University of Toronto, Shanghai Jiao Tong University and University of Michigan cooperated closely.

**Table 2:** The top 10 organizations with the most publications.

Rank	Organization	Documents	Citations	Average citation	Country/region
1	University of Sydney	91	4,594	50.48	Australia
2	Katholieke Universiteit Leuven	80	5,755	71.94	Netherlands
3	Tel Aviv University	70	8,260	118.00	Israel
4	Oregon Health and Science University	63	3,237	51.38	The United States
5	Radboud Universiteit Nijmegen	61	3,615	59.26	Netherlands
6	Capital Medical University	34	533	15.68	China
7	Tel Aviv Sourasky Medical Center	32	2,153	67.28	Israel
8	University of Waterloo	31	528	17.03	Canada
9	University of Washington	31	1,602	51.68	The United States
10	Universidade de São Paulo	27	280	10.37	Brazil

### 3.3 Analysis of journals and co-cited journals

**Figure 4:**
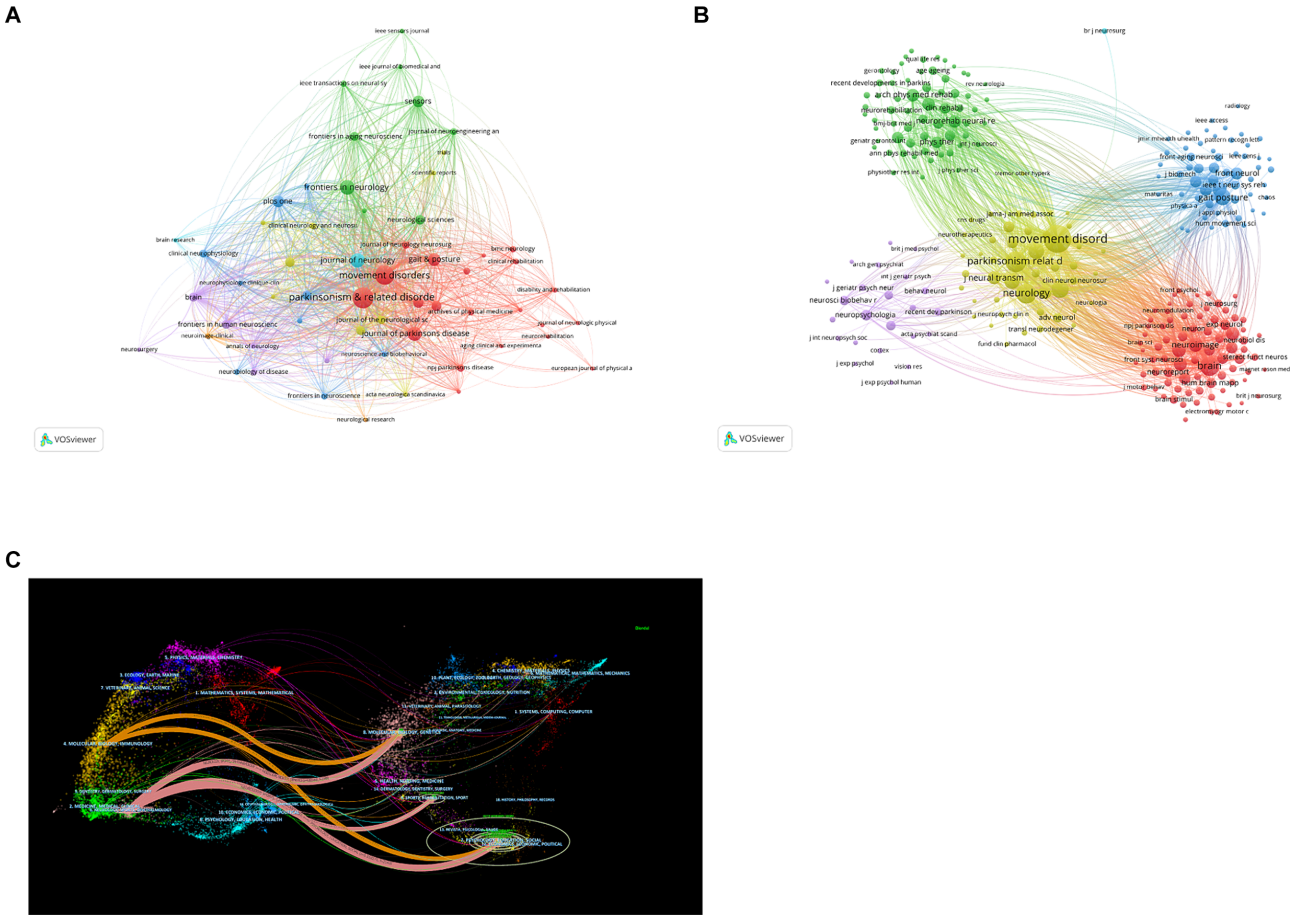
Analysis of journals and co-cited journals involved in studies related to PD-FOG. The minimum number of publications for the journals included in this map is five and the minimum number of citations for the co-cited journals included in this map is twenty. **(A)** The co-occurrence and clustering map of journals. **(B)** The co-occurrence and clustering map of co-cited journals. **(C)** The dual-map overlay of journals focusing on PD-FOG research.

In total, 301 academic journals have engaged in publishing articles focused on PD-FOG. Tables 3, 4 details the top 10 journals in terms of publication volume on PD-FOG research, as well as the 10 journals most frequently co-cited in this area. According to Table 3, *Parkinsonism & Related Disorders* leads in publications (*n* = 110), followed by *Movement Disorders* (*n* = 93) and *Frontiers in Neurology* (*n* = 55). *Movement Disorders* has the highest IF at 8.6. Remarkably, these journals exhibit significant positive citation relationships (Figures 4A,B). In addition to their large number of publications, *Movement Disorders* and *Parkinsonism & Related Disorders* are the two most frequently cited and co-cited journals, demonstrating their extensive influence and significant publishing potential in the field. Among the top 10 journals by citation count, eight have received more than 1,000 citations, and four have an IF above10. These data highlight the high quality of research and academic impact of the articles published in these journals. Figure 4C depicts a dual-map journals overlay, revealing the range of disciplines among journals focusing on PD-FOG research. A total of four different paths were identified. The orange paths indicate that studies published in Molecular/Biology/Genetics journals and Psychology/Education/Social journals often receive citations in publications from Neurology/Sports/Ophthalmology journals, while the pink paths indicate that studies published in Molecular/Biology/Genetics journals, Sports/Rehabilitation/Sport journals and Psychology/Education/Social journals were frequently referenced in studies published in Neurology/Sports/Ophthalmology journals.

**Table 3:** The top 10 journals with the most publications.

Rank	Journal	Documents	Citations	Average citation	IF	JCR	Country/region
1	Parkinsonism & Related Disorders	110	4,197	38.15	4.1	Q2	The United Kingdom
2	Movement Disorders	93	7,959	85.58	8.6	Q1	The United States
3	Frontiers in Neurology	55	784	14.25	3.4	Q2	Switzerland
4	Journal of Neurology	50	1882	37.64	6.0	Q1	Germany
5	Journal of Parkinson’s Disease	50	789	15.78	5.2	Q1	Netherlands
6	Gait & Posture	40	1,360	34.00	2.4	Q4	Ireland
7	Plos One	33	1,214	36.79	3.7	Q2	The United States
8	Sensors	33	831	25.18	3.9	Q2	Switzerland
9	Parkinson’s Disease	27	730	27.04	3.2	Q2	The United Kingdom
10	Neurorehabilitation and Neural Repair	24	988	41.17	4.2	Q2	The United States

**Table 4:** The top 10 co-cited journals with the most citations.

Rank	Journal	Citations	IF	JCR	Country/region
1	Movement Disorders	8,284	8.6	Q1	The United States
2	Parkinsonism & Related Disorders	3,369	4.1	Q2	The United Kingdom
3	Neurology	2,800	10.1	Q1	The United States
4	Brain	2,635	14.5	Q1	The United Kingdom
5	Journal of Neurology, Neurosurgery, Psychiatry	1847	11.1	Q1	The United States
6	Gait & Posture	1,515	2.4	Q4	Ireland
7	Journal of Neurology	1,333	6	Q1	Germany
8	Lancet Neurology	1,132	48	Q1	The United States
9	Plos One	915	3.7	Q2	The United States
10	Neuroimage	908	5.7	Q1	The United States

### 3.4 Analysis of co-authors

**Figure 5:**
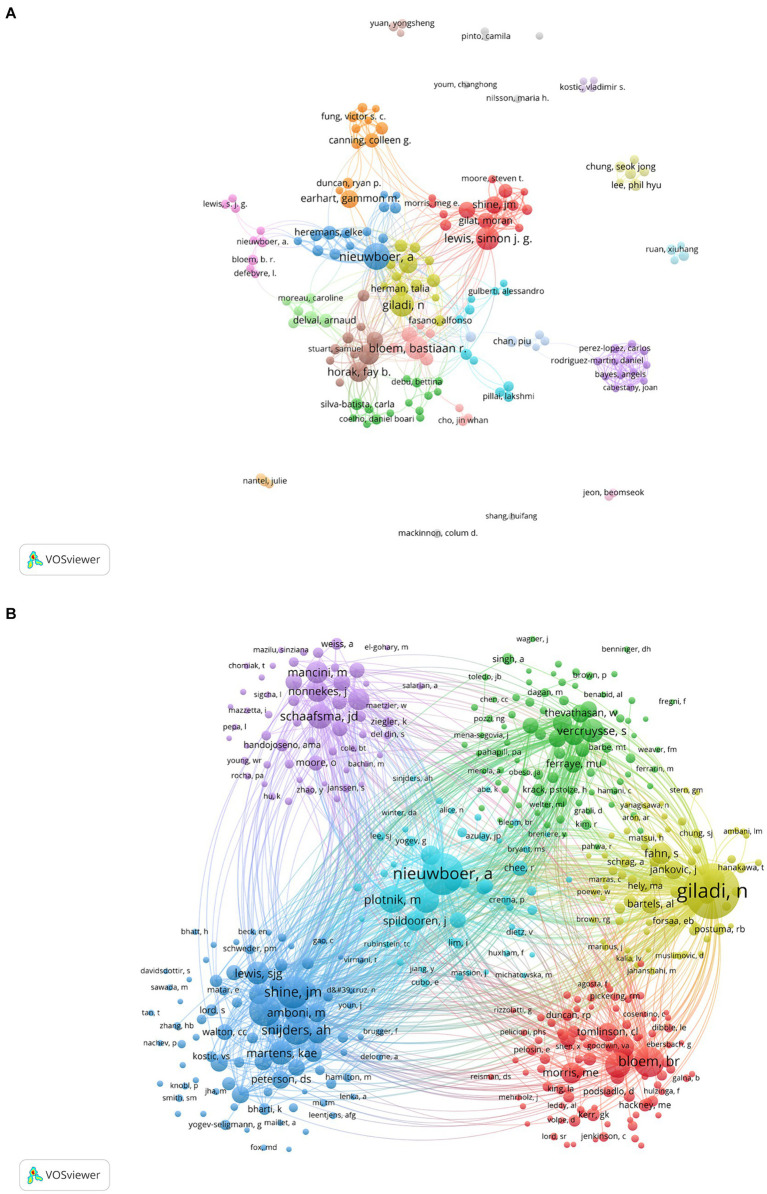
Analysis of core authors and co-cited authors involved in studies related to PD-FOG. **(A)** The co-occurrence and clustering map of core authors. **(B)** The co-occurrence and clustering map of co-cited authors with a minimum number of citations of 20.

In this field, a collective of 5,142 co-authors have made contributions. To identify the most influential contributors to PD-FOG studies over the past two decades, top 10 co-authors were arranged based on the number of their published articles (Table 5). Among the high-volume authors, Nieuwboer A was the most productive author with a total of 68 articles from 2000 to November 2023. After her, Giladi N ranked second with 54 articles and Lewis S.J.G ranked third with 53 articles. According to Price’s Law, the minimum publication volume for core authors is *m* = 0.749 × √Nmax. Nmax is the number of articles published by the authors who have published the most articles (Nmax = 68, *m* = 6.18 here) (Price, 1963), therefore core author here is defined as the author who has published at least seven articles and there were totally 123 core authors in our analysis. We visualized network maps of 123 core authors to illustrate their collaborative degree (Figure 5A). Notably, not only the authors in the same cluster have shown strong cooperation, but also the authors in different clusters have actively cooperated.

**Table 5:** The top 10 authors with the most publications.

Rank	Author	Documents	Citations	Average citation	Country/region
1	Nieuwboer A	68	4,506	66.26	Belgium
2	Giladi N	54	8,012	148.37	Israel
3	Lewis S.J.G	53	2,559	48.28	Netherlands
4	Bloem B.R	50	3,318	66.36	Netherland
5	Mancini M	42	1,421	33.83	The United States
6	Horak F.B	37	2,461	66.51	The United States
7	Hausdorff J.M	33	2,807	85.06	The United States
8	Earhart G. M	30	1,502	50.07	The United States
9	Shine J.M	28	2,270	81.07	The United States
10	Nutt J.G	27	813	30.11	The United States

As for co-cited authors, Giladi N ranked first with 1788 citations, followed by Nieuwboer A (1,039 citations) and Shine J.M (604 citations) (Table 6). Figure 5B presents a co-citation network map of co-cited authors with a minimum number of citations of 20, showing six different clusters, represented by Nieuwboer A, Giladi N, Bloem B.R, Shine J.M, Schaafsma J.D and Vercruysse S, respectively. There is active collaboration both within and between the various clusters. It’s worth noting that Nieuwboer A and Giladi N also rank high among co-authors, indicating they are influential researchers in the field.

**Table 6:** The top 10 co-cited authors with the most citations.

Rank	Co-cited author	Country/region	Citations
1	Giladi N	Israel	1788
2	Nieuwboer A	Belgium	1,039
3	Shine J.M	Australia	604
4	Nutt J.G	The United States	589
5	Bloem B.R	Netherlands	549
6	Snijders A.H	Netherlands	474
7	Plotnik M	Netherlands	431
8	Goetz C.G	The United States	387
9	Schaafsma J.D	Canada	362
10	Hausdorff J.M	The United States	355

### 3.5 Analysis of co-cited references

Co-cited references analysis plays a crucial role in uncovering the core knowledge base and significant articles from a broad spectrum of references, thereby enabling an in-depth exploration of advancements within the field (He et al., 2023). Table 7 displays the 20 most frequently co-cited references. ‘Freezing of gait: moving forward on a mysterious clinical phenomenon’, published by Nutt J.G, is the most frequently cited article (*n* = 153). This article summarized the clinical characteristics, physiological features of FOG, and its treatment methods, as well as discussed several existing hypotheses on the pathogenesis of FOG. Furthermore, ‘Freezing of gait: a practical approach to management’ attracted the second-highest co-citations (*n* = 81). This study explored therapeutic strategies for FOG, covering both medical and non-medical interventions, and proposed a practical management algorithm for FOG. The third most cited reference, ‘Gait-related cerebral alterations in patients with Parkinson’s disease with freezing of gait’, introduced imaging evidence of alterations both structurally and functionally within the midbrain motor area among PD-FOG patients, offering novel insights on studies in PD-FOG.

**Table 7:** The top 20 co-cited references.

Rank	First author	Year	Title	Journal	Counts
1	Nutt JG	2011	Freezing of gait: moving forward on a mysterious clinical phenomenon	Lancet Neurol	153
2	Nonnekes J	2015	Freezing of gait: a practical approach to management	Lancet Neurol	81
3	Snijders A.H	2011	Gait-related cerebral alterations in patients with Parkinson’s disease with freezing of gait	Brain	79
4	Ginis P	2018	Cueing for people with Parkinson’s disease with freezing of gait: A narrative review of the state-of-the-art and novel perspectives	Annals of Physical and Rehabilitation Medicine	72
5	Naismith S.L	2010	The Specific Contributions of Set-Shifting to Freezing of Gait in Parkinson’s Disease	Movement Disorders	61
6	Snijders A.H	2016	Physiology of freezing of gait	Annals of Neurology	59
7	Snijders A.H	2012	Freezer or non-freezer: Clinical assessment of freezing of gait	Parkinsonism & Related Disorders	58
8	Weiss D	2020	Freezing of gait: understanding the complexity of an enigmatic phenomenon	Brain	56
9	Shine J.M	2013	Exploring the cortical and subcortical functional magnetic resonance imaging changes associated with freezing in Parkinson’s disease	Brain	55
10	Giladi N	2009	Validation of the Freezing of Gait Questionnaire in Patients with Parkinson’s Disease	Movement Disorders	55
11	Almeida Q.J	2010	Freezing of gait in Parkinson’s disease: a perceptual cause for a motor impairment?	Journal of Neurology, Neurosurgery and Psychiatry	54
12	Perez-Lloret S	2014	Prevalence, Determinants, and Effect on Quality of Life of Freezing of Gait in Parkinson Disease	JAMA Neurology	54
13	Fling B.W	2014	Functional Reorganization of the Locomotor Network in Parkinson Patients with Freezing of Gait	Plos One	53
14	Martens K.A.E	2018	Predicting the Onset of Freezing of Gait: A Longitudinal Study	Movement Disorders	53
15	Spildooren J	2010	Freezing of Gait in Parkinson’s Disease: The Impact of Dual-Tasking and Turning	Movement Disorders	53
16	Mancini M	2017	The clinical significance of freezing while turning in parkinson’s disease	Neuroscience	50
17	Chee R	2009	Gait freezing in Parkinsons disease and the stride length sequence effect interaction	Brain	49
18	Martens K.A.E	2018	Evidence for Subtypes of Freezing of Gait in Parkinson’s Disease	Movement Disorders	48
19	Giladi N	2008	Understanding and treating freezing of gait in parkinsonism, proposed working definition, and setting the stage	Movement Disorders	48
20	Lewis S.J.G	2016	The Next Step: A Common Neural Mechanism for Freezing of Gait	Neuroscientist	48

**Figure 6:**
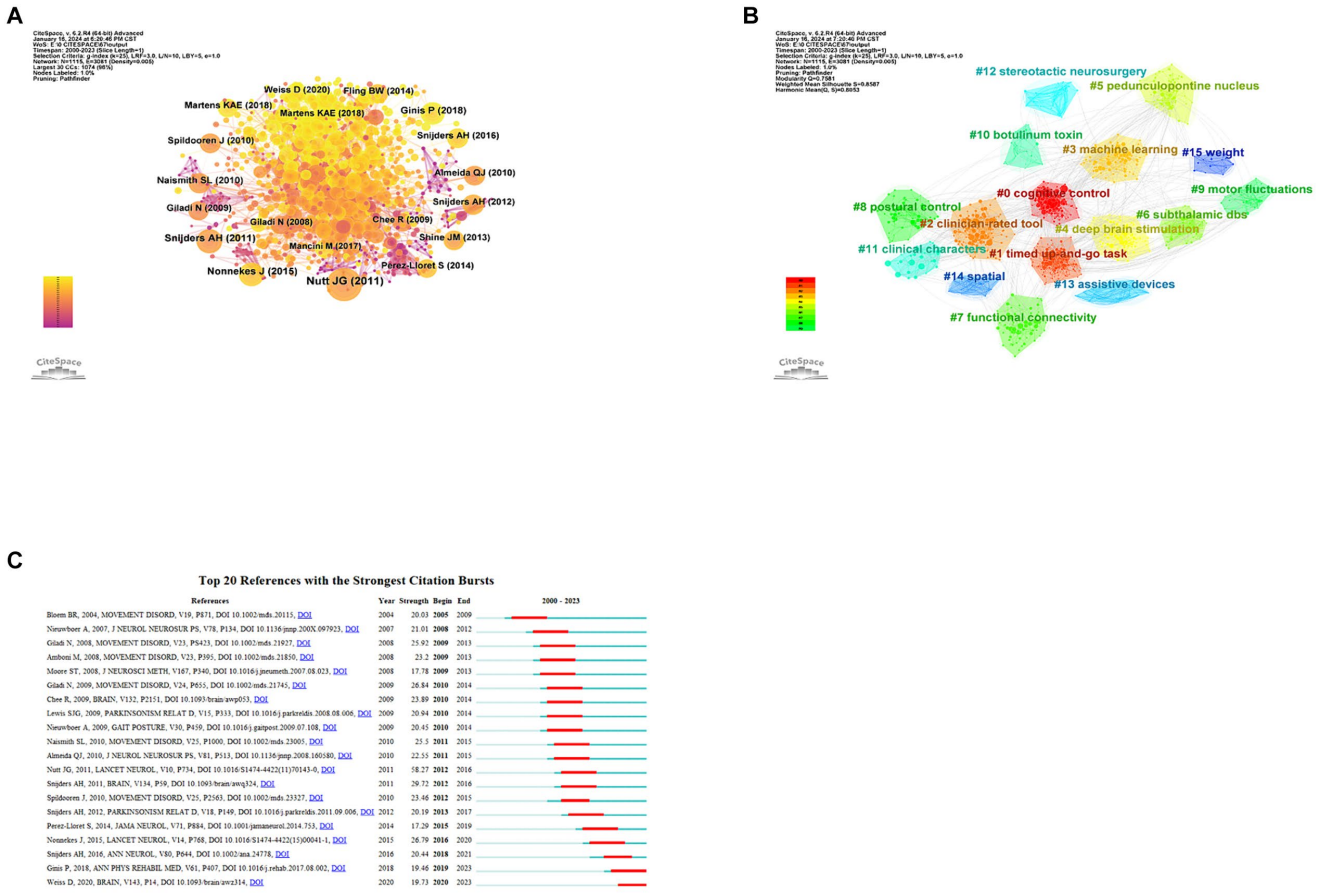
Analysis of co-cited references about PD-FOG. **(A)** The co-occurrence map of co-cited references about PD-FOG. **(B)** The clustering map of co-cited references about PD-FOG. **(C)** The top 20 references with the strongest citation bursts.

By conducting a cluster analysis of references, we can detect current research interests and anticipate future trends. From Figure 6A, it is evident that frequent citations occur mainly in recent literature, represented by yellow and orange nodes, indicating that the field is rapidly developing and attracting a great deal of scholar attention. The recent published literatures are representative and authoritative. The clustering map of co-cited references (Figure 6B) shows 15 clusters in total, among which the three most prominent are centered around the themes of ‘cognitive control’, ‘timed up-and-go task,’ and ‘clinician-rated tool’.

Citation burst analysis can identify which citations have surged over a period of time, identifying key studies that have had a significant impact on the academic field. Figure 6C reveals the top 20 references with the strongest citation bursts. Apparently, ‘Freezing of gait: moving forward on a mysterious clinical phenomenon’ has the strongest citation burst strength. The latest article with the strongest burst strength published in 2020 (Weiss et al., 2020), summarizes the research progress of complex determinants and pathophysiology of FOG. This article is still exhibiting burstiness. Another study with consistent burstiness is conducted by Pieter Ginis summarizing the effect of the cue method as a means of rehabilitation for PD-FOG (Ginis et al., 2018).

### 3.6 Analysis of keywords

**Figure 7:**
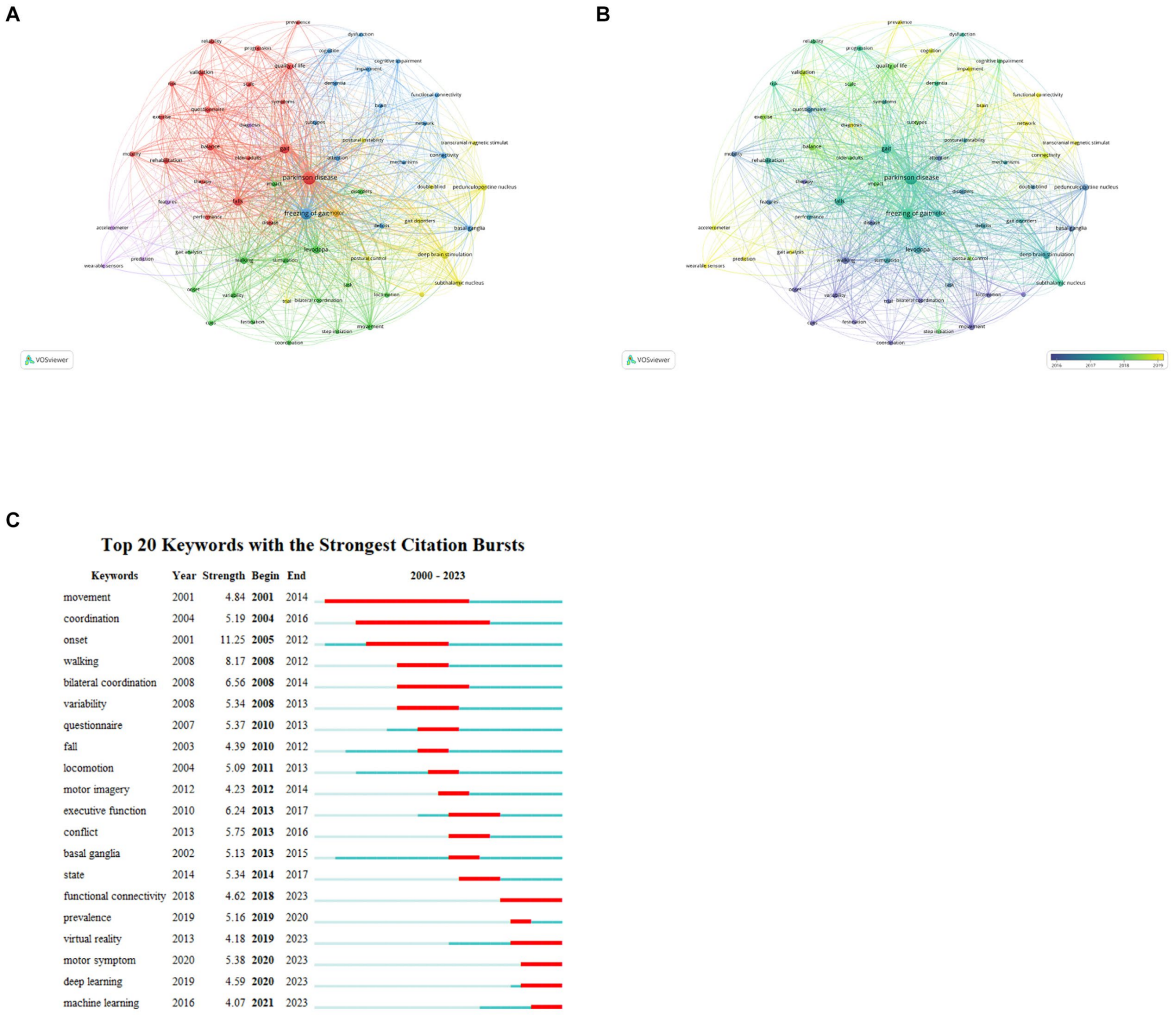
Analysis of keywords about PD-FOG. **(A)** The co-occurrence map keywords about PD-FOG, with a minimum number of occurrences of 30. **(B)** The overlay visualization map of keywords about PD-FOG, with a minimum number occurrences of 30. **(C)** The top 20 keywords with the strongest citation bursts.

The theme of an article is encapsulated by its keywords, which are instrumental in analyzing the research hotspots and frontiers of a specified field. Table 8 detailed the 20 keywords with highest frequency, each of which appeared at least 70 times. We filtered out keywords with more than 30 occurrences and clustered them using VOSviewer (Figure 7A). A total of six clusters were obtained, representing six diverse research directions. The largest cluster, cluster1 (red) centers around ‘Parkinson’s disease’, and encompasses keywords such as ‘gait’, ‘falls’, ‘balance’, ‘questionnaire,’ and similar terms. These keywords focus on impact of PD-FOG on patients’ activities of daily living and quality of life. Cluster 2 (green) mainly contains terms like ‘levodopa’, ‘walking’ ‘movement,’ and ‘variability’, concentrating on medication therapy and clinical phenomenon. With ‘freezing of gait’ as its core, cluster 3 (blue) primarily including ‘basal ganglia’, ‘connectivity’, ‘deficits’, ‘attention’, ‘dementia,’ and similar terms, is related to cognitive deficits and brain connectivity of PD-FOG. Cluster 4 (yellow) has 10 items, mainly covering ‘deep brain stimulation’, ‘subthalamic nucleus’, ‘transcranial magnetic stimulation’. These terms are related to neuromodulation of PD-FOG. Cluster 5 (purple) consists of ‘features’, ‘diagnosis’, ‘prediction’, ‘accelerometer,’ and ‘wearable sensors’, centering on diagnosis and prediction. Finally, cluster 6 (orange) distinctly defined by a sole keyword ‘motor’. The overlay visualization map of high-frequency (*n* > 30) reflects that ‘prediction’, ‘functional connectivity’, ‘wearable sensors,’ and similar keywords depicted in yellow nodes, are emerging research topics (Figure 7B). In Figure 7C, the 20 keywords demonstrating the highest intensity of citation bursts are highlighted, the top three are ‘onset’, ‘walking’ and ‘bilateral coordination’. ‘functional connectivity’, ‘virtual reality’, ‘motor symptom’, ‘deep learning’, ‘machine learning’ are the strongest keywords bursting in past 5 years, indicating these research topics have garnered significant interest and may become the forthcoming research hotspots in the field.

**Table 8:** The top 20 keywords with highest frequency.

Rank	Keyword	Counts
1	Parkinson disease	1,010
2	Freezing of gait	675
3	Gait	361
4	Falls	249
5	Levodopa	186
6	Deep brain stimulation	175
7	Balance	163
8	Walking	143
9	Motor	142
10	Quality of life	125
11	Questionnaire	113
12	Subthalamic nucleus	104
13	Movement	99
14	Pedunculopontine nucleus	95
15	Rehabilitation	92
16	Validation	88
17	Basal ganglia	82
18	Connectivity	82
19	Stimulation	76
20	Variability	70

## 4 Discussion

### 4.1 Basic information

In this study, we use Vosviewer and CiteSpace to analyze 1,340 articles related to PD-FOG that retrieved from WOSCC. Despite fluctuations, a notable trend of growth in the volume of publications in this field is clearly observed from 2020 to 2023. Since 2018, the annual publication volume has consistently exceeded 100, reflecting PD-FOG has gained extensive attention from scholars and was widely researched.

The top three countries that publish articles that the United States, China, and Australia, with the United States playing a central role in the international cooperation network. As a developed, high-income North American country, the USA has a relatively high incidence rate of PD globally. Additionally, the USA leads in fields like engineering and computer science, which fosters the advancement of interdisciplinary research. The use of advanced technologies enables researchers to explore PD-FOG mechanisms and treatment strategies more thoroughly. Furthermore, the USA boasts many top-tier research institutions and academic teams, which gather scientific talent. Our result reveals that at least half of the key contributing authors, co-cited authors and journals are from the United States, signifying the nation’s status as a center for authoritative research organizations and accomplished.

Meanwhile, the USA actively cooperated with other countries and have made important contributions to progress in the field of PD-FOG. In contrast, although China have published a large number of articles, its impact is relatively low, and there is a lack of cooperation with other countries. This may be the direction that China needs to work toward in the future. Regarding organizations, obviously, Tel Aviv University and Katholieke Universiteit Leuven are leaders among organizations performing research on PD-FOG, contributing considerable excellence in this field. As for authors, Nieuwboer A and Giladi N are two most influential scholars in the field. They all focused on assessing, quantifying, and improving frozen gait. Nieuwboer A primarily focused on analyzing the kinematic characteristics of PD-FOG (Nieuwboer et al., 2001, 2004; Alice et al., 2007), while Giladi N’s research encompassed construction and validation of the Freezing of Gait Questionnaire (FOGQ), the exploration of clinical characteristics of FOG and the study of medication treatments and their effectiveness for FOG (Giladi et al., 2000, 2001, 2009; Giladi, 2008).

In our journal analysis, *Parkinsonism & Related Disorders*, *Movement Disorders* and *Frontiers in Neurology* stand out for their large publication numbers and have received a considerable amount of citations in the co-citation analysis. Among them, *Movement Disorder*s has the highest IF. It aims at Parkinson’s disease and other movement disorders, enjoys widespread recognition and holds substantial influence in PD-FOG, making a good choice for researchers when submitting manuscripts. Moreover, the dual-map journals overlay reveals that research related to PD-FOG is mainly focused on the fields of life sciences, clinical medicine, rehabilitation science and social sciences. Future studies should concentrate more on interdisciplinary collaboration to advance the academic field.

### 4.2 Research hotspots

Analyzing the co-cited literature and keywords offers insights into the foundational themes and crucial aspects of contemporary studies (Xu et al., 2022), also helps identify the hotspots and trends in a specific field. In our study of PD-FOG, we have identified four major research subjects around PD-FOG: clinical kinematic, diagnosis and evaluation methods, activities of daily living and quality of life, cognitive deficits and brain connectivity, alongside therapy and rehabilitation.

#### 4.2.1 Clinical kinematic features

Basic clinical features of FOG include its frequent occurrence during gait initiation, walking, or turning, manifesting as trembling in place, complete freezing, or shuffling forward in small steps (Nutt et al., 2011). Besides, PD patients with FOG display a series of unique clinical signs in their gait. Nieuwboer A et al. demonstrated that freezing stems from a complex problem: the growing incapacity to maintain stride length and a dysregulation in the rhythm of walking (Nieuwboer et al., 2001). It has been widely accepted that FOG manifests as an entirely episodic, acute occurrence, with patients’ gait returning to normal after navigating past the motor impediment. However, the study by Hausdorff J. M firstly demonstrated that PD patients with FOG suffer from persistent gait disturbance. Specifically, these patients show marked difficulties in regulating the timing differences between strides and keeping a consistent walking rhythm (Hausdorff et al., 2003). This finding stimulated scholars to explore the kinematic characteristics of PD-FOG patients. A gait analysis study using a split-belt walking for PD patients with and without FOG, showed that stride time variability and stride time asymmetry increased in freezers, supporting the theory that FOG is associated with both gait imbalances and timing irregularities (Nanhoe-Mahabier et al., 2013). Other studies have also showed that PD patients with FOG exhibit greater variability in step length (Thevathasan et al., 2012; Virmani et al., 2018), step time (Gilat et al., 2013) and foot strike, which may increase the risk of falls due to instability (Shah et al., 2018). Besides, some studies have indicated that PD patients facing FOG have notable uncoordinated gait and gait asymmetry on both sides (Plotnik et al., 2005, 2008; Plotnik and Hausdorff, 2008), with this bilateral incoordination even becoming more distinct during complex gait tasks (Peterson et al., 2012). And the sequence effect, a common phenomenon in PD characterized by gradual step to step reduction, is also regarded as a potential relevant factor to FOG in study by Chee et al. (2009). A longitudinal study noted that, relative to those without FOG, spatiotemporal gait parameters in PD subjects developing FOG and in those with FOG declined faster. The progression speed in spatiotemporal gait parameters effectively predicted the conversion to FOG (Virmani et al., 2023). Overall, FOG is the result of combination of multiple deficiencies in gait features. These findings provided some theoretical basis for the pathogenesis of PD-FOG, but the evidence is insufficient as most of these are small sample studies. It is necessary to conduct more validation studies in the future, so that the kinematic features of PD-FOG could be utilized in clinical diagnosis, therapy, and rehabilitation.

#### 4.2.2 Diagnosis and evaluation methods

Accurately diagnosing and assessing FOG presents challenges for medical professionals. Many patients are unaware they have encountered FOG, unconsciously deny the condition due to unfamiliarity with its appearance (Snijders et al., 2008). The current definition of FOG is “a brief, episodic absence or marked reduction of forward progression of the feet despite the intention to walk” (Giladi and Nieuwboer, 2008). It provides the theoretical basis for medical professionals to identify FOG through clinical observation. By adding some challenging tasks to patients with PD-FOG while they are walking such as crossing a narrow space, turning around and holding something on the hand, could increase the probability of detecting FOG in a clinical environment (Snijders et al., 2008). Beyond that, histories or questionnaires can also indicate the presence and severity of the phenomenon. Movement Disorder Society-sponsored revision of the Unified Parkinson’s Disease Rating Scale covers questions for identifying and assessing the severity of FOG. However, these questions are too simple and rough to assess the degree for FOG in a professional evaluation, also lacking specialized validation (Goetz et al., 2008). Giladi N et al. constructed the original freezing of gait questionnaire (FOG-Q) and validated it, offering an effective screening tool for FOG (Giladi et al., 2000, 2009). The other validated questionnaire is new freezing of gait questionnaire (NFOG-Q), which is developed from and largely based on its previous version (Nieuwboer et al., 2009). Although there have been more scales emerging, these two questionnaires are most widely used and recognized in professional studies. Given FOG’s episodic nature, heterogeneous manifestation, it’s difficult to detect and manage it in clinical environment effectively. Wearable sensors, combined with statistical approaches for the automatic detection and prediction of FOG, have become promising for achieving consistent, objective monitoring over time and for use in daily life These methods show significant accuracy and precision, enabling the detection and assessment of FOG in real-life settings, and potentially guiding personalized rehabilitation strategies (Capecci et al., 2016; Silva de Lima et al., 2017; Mancini et al., 2021; May et al., 2023).

#### 4.2.3 Activities of daily living and quality of life

PD can result in activities limitations and balance deficit this is especially evident in individuals with early-onset Parkinson’s disease (EOPD) who suffer from FOG (Giladi et al., 2009; Korkusuz et al., 2023). The occurrence of FOG significantly increases the risk of falls among PD patients, which may result in the need for nursing home placement, thereby limiting their activities and adding to the burden on caregivers (Schrag et al., 2006; Kerr et al., 2010; Tan et al., 2011; Okuma et al., 2018). Even among individuals with early-onset PD, it’s apparent that daily physical activity is reduced in individuals facing freezing episodes versus those without such episodes (Terashi et al., 2022). Such differences might originate from the fear of falling, causing patients to loss postural control and undermining their confidence in carrying out daily activities (Adkin et al., 2003; Brozova et al., 2009). Reduced mobility not only leads to a loss of independence but also strips patients of their social interactions, resulting in significant isolation for some individuals. In fact, Courtney C. W et al. demonstrated that, even considering the impact of other key variables, FOG-Q scores independently predict the scores of health-related quality of life (HRQoL) in the earlier clinical stages of PD (Perez-Lloret et al., 2014; Walton et al., 2015b). Beyond the indirect effect of FOG on mobility, FOG also leads to a decline in patients’ quality of life through impacts on bodily discomfort, activity of daily living (ADL), emotional, communication and cognition (Moore et al., 2007). Multiple tools designed to assess daily living function and quality of life, such as 39-item Parkinson’s Disease Questionnaire (PDQ-39), 36-Item Short Form Health Survey (SF-36), Europe Quality of Life Questionnaire-visual analogue scale (EQ-VAS) and the Europe Quality of Life Questionnaire-5D (EQ-5D), are available to quantify the diverse impact of FOG on patients’ daily life (Jenkinson et al., 1999; Schrag et al., 2000; Winter et al., 2010; Tu et al., 2017; Zhao et al., 2021).

#### 4.2.4 Cognitive deficits and brain connectivity in FOG

Given the harm of FOG, it is crucial to clarifying the neural mechanisms behind its appearance. Currently, scholars are highly focused on cognitive deficits and brain connectivity in PD-FOG. The execution of gait requires the involvement of cognitive system and motor system (Kelly et al., 2012). PD patients can display cognitive deficits at an early stage, including memory impairment, visuospatial dysfunction and executive dysfunction (Yarnall et al., 2014). These cognitive deficits are regarded as potential factors contributing to gait disorders in PD patients (Kelly et al., 2012). They might cause FOG through complex mechanisms (Vercruysse et al., 2012; Walton et al., 2015a; Yao et al., 2017; Gan et al., 2023). Worthwhile, FOG become more evident during the cognitive tasks (Monaghan et al., 2023). Nevertheless, most of studies are within small cohorts. A large cohort studies of PD demonstrate that after controlling of covariates, the difference between PD patients with and without FOG was not significant, indicating FOG and cognitive impairments may be two parallel process related to motor disease severity (Morris et al., 2020). To verify the causal correlation between FOG and cognition deficits necessitates further large-scale cohort studies.

In recent years, the emphasis of PD-FOG related fields is gradually moving to the exploration of brain connectivity features. Structural connectivity changes were explored by diffusion tensor imaging (DTI) and it was found that PD patients with FOG had diffuse white matter lesions, decreased structural connectivity between motor, cognitive, and limbic structures involved in advanced gait control, and also between bilateral cerebral hemispheres, which may be the basis of FOG (Fling et al., 2013; Vercruysse et al., 2015; Pietracupa et al., 2018; Jin et al., 2021). Substantial functional magnetic resonance imaging (fMRI) research on FOG show suggests that, unlike PD patients without FOG, those with FOG show abnormalities in functional activation and connectivity in some brain areas, such as the frontal and parietal lobes, sensorimotor pathways, subcortical areas (especially in the midbrain), and cerebellum (Bartels and Leenders, 2008; Snijders et al., 2011; Shine et al., 2013a; Zhou et al., 2018; Potvin-Desrochers et al., 2019; Jung et al., 2020; Lench et al., 2020, 2021; Li et al., 2021). Remarkably, it appears that the right hemisphere’s circuitry is more profoundly affected than the left’ s in individuals with PD-FOG (Fling et al., 2013). Based on those studies, investigations into FOG treatments have become more targeted. Therefore, we think that fMRI techniques may help us to further clarify the mechanism of FOG and find new breakthroughs in targeted treatment.

#### 4.2.5 Therapy and rehabilitation of PD-FOG

Early treatment and rehabilitation training are crucial for the prognosis of patients with PD-FOG. In drug treatments for PD-FOG, the preferred drug regimen is levodopa replacement therapy, which is especially effective for “off”-related FOG but has limited improvement for other subtypes of FOG (Schaafsma et al., 2003; Fietzek et al., 2013). In contrast, Levodopa-Carbidopa Intestinal Gel (LCIG), by altering the drug delivery method, maintains steadier dopamine levels in the bloodstream, thereby improving different subtypes of FOG (Vijiaratnam et al., 2018). As for dopamine-induced fFOG, reduction in levodopa seems to be a good choice (Espay et al., 2012). Other drugs, such as Rotigotine (Ikeda et al., 2016), selegiline (Iijima et al., 2017), rasagiline (Zhang et al., 2016; Cibulcik et al., 2016), istradefylline (Iijima et al., 2019), methylphenidate (Moreau et al., 2012), and botulinum toxin (Giladi et al., 2001) have shown some potential in the improvement on PD-FOG, but their effect vary in different studies (Fernandez et al., 2004; Gurevich et al., 2007; Espay et al., 2011). More large-scale validation studies are needed to provide evidence. In terms of non-pharmacological therapies, subthalamic nucleus deep brain stimulation (STN-DBS) is widely recognized as a routine surgical treatment (Di Rauso et al., 2022). Pedunculopontine nucleus (PPN) is a promising but experimental surgical target (Thevathasan et al., 2018). In addition, in some research on treating PD-FOG, spinal cord stimulation (SCS) has displayed huge potential (de Lima-Pardini et al., 2018). For patients who experience poor medication outcomes and refuse invasive surgeries, non-invasive neuromodulation is an alternative option. tDCS and rTMS, two common non-invasive neuromodulation techniques, effectively modulate the excitability of the targeted cortex regions, contributing to the alleviation of Parkinson’s disease symptoms. These methods have garnered scholarly attention due to their low cost and non-invasiveness, especially research dedicated to addressing FOG. According to some studies, stimulation with tDCS or rTMS targeted to primary motor cortex (PMC) or left dorsolateral prefrontal cortex (DLPFC) could effectively ameliorate PD-FOG (Lee et al., 2014; Valentino et al., 2014; Dagan et al., 2018; Mi et al., 2019). Even without altering the stimulation targets, optimizing the stimulation parameters may lead to surprising effects. Jinmei Sun et al. firstly applied accelerated high-dose TBS (ahTBS) protocol targeting to PMC and achieve an unprecedented efficiency in the literature describing treatment of FOG (Sun et al., 2021). Apart from this, noninvasive vagus nerve stimulation (VNS) also showed the effect in improving FOG in study by Mondal et al. (2019). To date, intervention training methods for FOG rehabilitation have predominantly concentrated on improvement of cognitive function and exercise training such as resistance training and treadmill training to overcome freezing events (Peterson et al., 2016; Rutz and Benninger, 2020). Some studies have shown that cues can also be used to improve FOG (Cao et al., 2020; Graham et al., 2023; Klaver et al., 2023b; Zoetewei et al., 2024), and it is worth trying for long-term rehabilitation.

### 4.3 Future research trends

Based on keyword overlay analysis and burst keyword analysis, we identified a number of topics that are on the research boom and are likely to remain hot research trends in the future, including functional connectivity, virtual reality, deep learning, and machine learning.

#### 4.3.1 Functional connectivity

Recently, research on functional connectivity has demonstrated significant potential in uncovering the pathological mechanisms and improving the diagnostic evaluation of PD-FOG. Through fMRI, researchers have identified dysfunctional connectivity in widespread cortical and subcortical regions (Song et al., 2021). Compared to MRI, EEG is faster, more comfortable, and less expensive, making it especially appropriate for monitoring patients’ brain function in outpatient clinics. Resting-state EEG research has shown that patients with PD-FOG exhibit greater frontoparietal functional connectivity than healthy controls (HC), and this connectivity intensifies with the severity of FOG, further supporting the link between FOG and cognitive impairments (Bayot et al., 2022). Various studies have discovered notable EEG features. With the progression of PD and FOG severity, the patient’s EEG network connectivity strengthens over a wide range of brain frequencies (Asher et al., 2021). Patients with FOG generally display reduced bereitschaftspotential (BP) amplitudes, suggesting an impairment in their gait preparation (Brugger et al., 2020; Marquez et al., 2023).

Investigations into PD patients who underwent deep brain stimulation (DBS) have identified that freezing episodes are linked to pathological abnormalities in subthalamic nucleus (STN) activity, including increased β and θ rhythms (Toledo et al., 2014; Georgiades et al., 2019). Additionally, low-frequency cortical-subthalamic decoupling is observed in the hemisphere with reduced striatal dopaminergic innervation in patients with FOG (Pozzi et al., 2019), reinforcing the significant role of the STN in freezing episodes. An EEG study combined with a series of timed up-and-go tasks found that, compared to walking, freezing episodes were related to a notable increase in theta band power in the central and frontal leads. The shift from normal walking to FOG was associated with elevated theta frequency coupling between these leads and increased cross-frequency coupling in the central lead (Shine et al., 2014). This specific EEG activity pattern indicates the feasibility of using EEG technology to predict FOG before it manifests. Magnetoencephalography (MEG), due to its high temporal resolution, can also be used to study neuronal activity and functional connectivity between different brain regions (Salmelin and Baillet, 2009). While MEG has been utilized to study PD-related neurophysiological characteristics, its use in FOG studies is still lacking (Boon et al., 2019). Future scholars may consider pursuing more extensive studies in this field.

Overall, researches on functional connectivity not only provide new perspectives on understanding the pathological mechanisms underlying PD-FOG but also have the potential to provide neuroimaging biomarkers for early diagnosis and treatment evaluation. Future trends should focus on utilizing technologies like EEG and MEG to further explore dynamic changes in brain functional connectivity, develop new neurobiomarkers, and enhance early diagnosis and personalized treatment strategies.

#### 4.3.2 Virtual reality

In recent years, virtual reality (VR) technology has shown great potential in the diagnosis and treatment of FOG. When inducing FOG in clinical and laboratory settings is challenging, VR technology offers a first-person perspective, simulating real-life movement situations to trigger FOG. This not only reduces the potential danger to subjects but also creates more effective conditions for inducing FOG, thus enhancing experimental techniques (Bluett et al., 2019). The study by Amir Besharat et al. demonstrates that virtual doorway and hallway environments can induce kinematic changes associated with FOG episodes, which are consistent with forward falls commonly observed during actual FOG episodes (Besharat et al., 2022). Studies combined with the VR paradigm have found that patients with PD-FOG have significant deficits in conflict resolution, visuospatial processing (Matar et al., 2013), motor initiation, and inhibition (Georgiades et al., 2016). In addition, VR combined with MRI provides insights into the functional connectivity mechanisms behind FOG. The study by Shine JM et al. found that patients experiencing FOG exhibit functional decoupling between the basal ganglia network and the cognitive control network in each hemisphere. This finding supports the hypothesis that freezing behavior in Parkinson’s disease stems from impaired communication between complementary yet competing neural networks (Shine et al., 2013b). Gilat M et al. simulated the turning process through VR and found that an increased propensity toward stopping in FOG, combined with reduced sensorimotor integration, might explain the neurobiological foundation of FOG during turning (Gilat et al., 2015). Compared with conventional resting-state fMRI, this research approach allows for a more thorough exploration of the specific brain network activity in FOG patients during movement. Studies have shown that virtual reality training as a rehabilitation method can improve gait and balance impairments and reduce the occurrence of FOG episodes in patients (Killane et al., 2015; Nuic et al., 2018). It can be expected that the potential application of VR in FOG research will be further expanded with its continuous progress in the future.

#### 4.3.3 Deep learning and machine learning

Monitoring and predicting the freezing of gait (FOG) is a crucial part of future research in this field. Currently, scholars have begun to utilize deep learning technology for evaluating and diagnosing FOG. Different deep learning models have been developed to analyze and process the data obtained by wearable devices, enabling the detection of FOG (Bikias et al., 2021; Borzì et al., 2023; Klaver et al., 2023a; Yang et al., 2024). However, the sample size of these studies is small, and more experimental data are needed to support their generalization in both home and healthcare settings. Machine learning technology also shows potential in wearable device data processing. Researchers have successfully implemented real-time detection of early-stage FOG by analyzing gait characteristics in both time and frequency domains (Arami et al., 2019; Zhang et al., 2020; Borzì et al., 2021). However, the accuracy of existing machine learning analysis in predicting FOG is not ideal. One study showed that machine learning models are able to make predictions with sufficient accuracy before FOG actually occurs, but dopaminergic therapy can alter the gait pattern before FOG, thereby affecting the effectiveness of the algorithm (Borzì et al., 2021). To improve the utility of the prediction model, it is necessary to increase the heterogeneity of participants by including patients with different drug regimens and fluctuations in drug efficacy, to validate and optimize the algorithm, thereby developing FOG monitoring models that are more suitable for daily life. In summary, the development of an optimal FOG monitoring algorithm model that takes into account both accuracy and specificity is still an important direction for future research by combining deep learning and machine learning technologies.

## 5 Limitations

There are still several limitations in our study. Firstly, we only included articles from WOSCC, which led to the possibility that we may overlook relevant articles from other databases. However, given that the WOSCC is widely used as a basic data source for bibliometric analysis and providing sufficient information needed for bibliometric analysis (Li et al., 2018), we have reason to believe that our results are convincing. Secondly, we only included articles written in English, which may result in missing some important documents written in other languages. Finally, due to the delay in citation, it is possible to miss recently published articles.

## 6 Conclusion

Our results reveal a consistent annual growth in publications related to PD-FOG over the past two decades, maintaining a stable high output since 2018. This trend indicates a promising research landscape in the field of PD-FOG. In this field, the United States holds a leading position, with Nieuwboer A and Giladi being two of the most influential researchers. Over the past two decades, the research hotspots for PD-FOG have primarily encompassed the kinematic characteristics, diagnosis and detection, cognitive deficits and neural connectivity, as well as therapy and rehabilitation of PD-FOG. It is anticipated that topics including functional connectivity, virtual reality, deep learning and machine learning will continue to be focal points of future research.

## Data Availability

The raw data supporting the conclusions of this article will be made available by the authors, without undue reservation.
